# Assessing the effect of child’s gender on their father–mother perception of the PedsQL™ 4.0 questionnaire: an iterative hybrid ordinal logistic regression/item response theory approach with Monte Carlo simulation

**DOI:** 10.1186/s12955-020-01601-y

**Published:** 2020-10-21

**Authors:** Marziyeh Doostfatemeh, Seyyed Mohammad Taghi Ayatollahi, Peyman Jafari

**Affiliations:** Department of Biostatistics, Shiraz University of Medical Sciences, Shiraz, Iran

## Abstract

**Background:**

This study aimed at investigating the possible confounding effect of children’s gender on the parents’ dyads perception of their child HRQoL at both item and scale levels of PedsQL^TM^4.0 questionnaire.

**Methods:**

The PedsQL™ 4.0 Generic Core Scales were completed by 573 children and their father-and-mother dyads. An iterative hybrid ordinal logistic regression/item response theory model with Monte Carlo simulation was used to detect differential item functioning (DIF) invariance across mothers/fathers and daughter/sons.

**Results:**

Assessing DIF across mother–daughter, father–daughter, mother–son, and father–son dyads revealed that although parents and their children perceived the meaning of some items of PedsQL^TM^4.0 instrument differently, the pattern of fathers’ and mothers’ report does not vary much across daughters and sons.

**Conclusion:**

In the Persian version of PedsQL^TM^4.0, the child’s gender is not a confounding factor in the mothers’ and fathers’ report with respect to their daughters’ and sons’ HRQoL. Hence, paternal proxy-reports can be included in studies, along with maternal proxy-reports, and the reports can be combined short of concerning children gender, when looking at parent–child agreement.

## Background

The inclusion of multiple informants in the field of health-related quality of life (HRQoL) of children has become the norm in clinical research practice [1]. Child’s self-report and fathers’ and mothers’ proxy-report are the most important sources of information when assessing the children’s HRQoL [2, 3]. Agreement between self- and proxy- ratings continues to be a controversial issue in pediatrics HRQoL studies [1, 4]. It was shown that child-parent agreement could be affected by child characteristics, such as age, sex and health condition [5, 6]. They have also indicated that parents often underestimate HRQoL for sick children, but they tend to rate healthy individuals upper than the children do themselves [1, 4]. However, the potential influence of the child’s gender has been rarely assessed in the literature, especially with respect to which of the parents are selected as a proxy respondent. In one of our recent studies, the potential interchangeability of the parent dyads in reporting children’s HRQoL was assessed on both item and scale levels of the PedsQL™ 4.0 instrument [7]. The study showed that parent–child agreement was not affected by the parents’ gender, but the discrepancies between parents and children regarding the child’s gender was not taken into account, which could have affected their report. A literature review in the field of child HRQoL indicated that daughters and sons had different relationships with each of their parents; it also showed that fathers and mothers had different perspectives for their child’s HRQoL [4]. Therefore, it is not easy to distinguish how far item rating of fathers and mothers is linked to their child’s gender [8]. Regarding the results of several studies, it could be hypothesized that mother/daughter and father/son dyads might be interesting subgroups to analyzes their influence on the interchangeability of parent proxy-reports about their children’s HRQoL [8–10].

Although the agreement between the children’s and their parents’ perception regarding the children’s HRQoL has been investigated at the item and scale levels [11–13], it has never been evaluated at item level of PedsQL™ 4.0 and no other instrument by simultaneously considering children’s and parents’ gender. According to a systematic review, the PedsQL™ 4.0 questionnaire is the most widely used instrument for measuring HRQoL amongst children and adolescents [14]. Therefore, the present study aimed to assess the effect of child’s gender on their father–mother perception of their child’s HRQoL on both item and scale levels of the generic PedsQL™ 4.0. In other words, we attempted to evaluate the measurement invariance of this instrument among daughter–mother, son–mother, daughter–father and son–father dyad (assessing in the item level) and the discrepancy (assessing in the scale level), to clarify how a child’s gender can affect the agreement between fathers and mothers.

It should be mentioned that evaluating the agreement amongst informants regarding their perception on child’s HRQoL is currently in transition from classic approaches (e.g. calculating the inter-class correlation or comparing the means) to adapt more modern methods, such as differential item functioning (DIF) analysis. DIF analysis examines whether or not people in different groups respond consistently to a particular item within a scale after controlling the underlying construct measured by the scale. There are two types of DIF: uniform and non-uniform. Uniform DIF is evident when the difference in item response probabilities is constant across complete construct domains. Non-uniform DIF occurs when the direction of DIF differs in various parts of the scale [15]. Hence, the results of this study can provide further evidence on comparability of HRQoL scores across different informants in child self-reports and parent proxy-reports of the PedsQL™ 4.0, using the iterative hybrid ordinal logistic regression/item response theory (OLR/IRT) approach.

## Methods

### Participants and instrument

The participants comprised of Iranian secondary school children from four educational districts, with diverse socioeconomic backgrounds from Shiraz, a major metropolitan city in southern Iran, along with their mothers and fathers. A two-stage cluster random sampling method was used for the selection process. Out of 60 secondary schools in each district, four were chosen at random (first stage). In the next step, a simple random sampling technique was used to choose two classes from each school by random number table. Then, all the children in the selected classes were automatically taken as samples in the second stage.

The child and parent-report of the Persian version of the PedsQL™ 4.0 Generic Core Scales which was translated and validated previously in Iran [16] made a questionnaire that was filled out by the children and their mothers and fathers. A trained researcher clarified the objective of this survey and distributed a set of documents among them, containing the child’s self-report, two parents’ proxy-report, and parents’ informed consent form. The children were asked to take the documents to their parents.

Parents and their children filled out the questionnaires at home and returned them to the research team. Out of the 950 distributed triplet questionnaires in 32 classes within 16 secondary schools, 573 were filled out completely, with the overall return rate of 60%. (No more than 5% missing item response was considered acceptable; it provided two students who were excluded from the analysis). In the final sample, 281 (49%) male and 292 (51%) female students with their parents were included. The study was approved by the local ethics committee of Shiraz University of Medical Sciences. The mean ± standard deviation of the fathers’, mothers’, boys’ and girls’ age were 45.6 ± 6.1, 39.9 ± 6.4, 14.48 ± 1.31 and 14.42 ± 1.58 years, respectively. The characteristics of the participants are presented in Table 1.

**Table 1:** Sociodemographic characteristics of the study population

Children	573
Daughter	281 (49%)
Age (mean ± SD)	14.42 ± 1.58
Son	292 (51%)
Age (mean ± SD)	14.48 ± 1.31
Grade	
Guidance school	305 (53%)
High school	268 (47%)
Mother	573
Age (mean ± SD)	39.9 ± 6.4
Education level	
Academic	310 (54%)
Nonacademic	263 (46%)
Father	573
Age (mean ± SD)	45.6 ± 6.1
Education level	
Academic	342 (60%)
Nonacademic	231 (40%)
Number of children (mean ± SD)	2.6 ± 1.1

*SD* standard deviation

The PedsQL™ 4.0 is a 23-item generic instrument, which consists of four scales including physical, emotional, social and school functioning (An eight-item scale and the three five-item scales). The participants responded to the items on a five-point Likert scale (0 = never a problem, 1 = almost never a problem, 2 = sometimes a problem, 3 = often a problem, and 4 = almost always a problem). The PedsQL™ 4.0 scoring protocol has reversed-scored items in a way that the higher scores indicate lower HRQoL.

### Statistical analysis

#### Differential item functioning analysis with iterative hybrid OLR/IRT approach

In this study, iterative hybrid OLR/IRT approach was implemented in the R package ‘‘*lordif*’’; it was used to examine DIF across daughters/sons and mothers/fathers in PedsQL™ 4.0 questionnaires [17]. In the DIF analysis through the OLR/IRT approach, along with providing statistical tests to identify the items exhibiting uniform and non-uniform DIF, the different magnitude and impact measures were also obtained to quantify the magnitude of DIF. The special feature of this approach is the usage of trait variable for matching rather than the observed scale score for the traditional OLR. OLR/IRT uses an iterative procedure to detect the DIF items by purifying trait score estimation during the analysis. At first, the algorithm fits a graded response model (GRM) [18] to obtain trait estimates. After that, a series of nested OLR models was fitted to detect the DIF items based on the OLR model criterion, conditioning on the estimated trait score which were obtained at the previous stage. Then, we refitted the GRM to obtain the revised trait estimate that accounts for *just* items identified with DIF in the former step. In the following stage, new DIF items are flagged again, and the results are compared with previous ones. If the same items are flagged, the analysis is stopped, but if different items are identified, we iterate the analysis until the discovered DIF and non-DIF items become the same as the ones detected in the previous run (for more details refer to Choi et al. [17]).

It is notable that the three nested OLR models which are responsible for identifying DIF items can be written, respectively, as:

$$\begin{aligned} & Model \,1{:}\, logit\, P\left( {Y_{i} \ge k} \right) = \alpha_{k} + \beta_{1} \times trait \\ & Model \,2{:}\, logit\, P\left( {Y_{i} \ge k} \right) = \alpha_{k} + \beta_{1} \times trait + \beta_{2} \times group \\ & Model \,3{:}\, logit\, \left( {Y_{i} \ge k} \right) = \alpha_{k} + \beta_{1} \times trait + \beta_{2} \times group + \beta_{3 } \times trait \times group \\ \end{aligned}$$

where $P\left( {Y_{i} \ge k} \right)$ is the probability of response in category *k* or higher of the item *i, α*_*k*_ is the intercept term which depends on the *k*th category of item *i*, *β*_1_ represents the effect of the trait (e.g. emotional functioning), *β*_2_ shows the effect of the group (fathers/mothers and daughters/sons), and *β*_3_ indicates the interaction effect between trait and group. Uniform DIF could be detected by comparing the log-likelihood values of Models 1 and 2 (i.e. β_2_ ≠ 0) and non-uniform DIF could be tested by comparing the log-likelihood values of Models 2 and 3 (i.e. β_3_ ≠ 0). Differences in the value of log-likelihoods are compared to the Chi-square distribution with one degree of freedom.

Since statistical power for testing uniform and non-uniform DIF is highly dependent on the sample size, a slight difference in the log-likelihood of the nested models can be statistically significant if there is a sufficiently large sample. In response to this concern, we used the McFadden [19] pseudo-R^2^ estimate [20] to quantify the magnitude of DIF and determine the clinical importance of DIF items. In most traditional analyses, classifying DIF is based on Zumbo guidelines (R^2^ < 0.13 as negligible, R^2^ between 0.13 and 0.26 as moderate and R^2^ > 0.26, as large) [21], but in this approach a Monte Carlo simulation-based procedure derives the thresholds or empirical criteria to determine whether the items have DIF, based on Type-I error rates empirically found in the simulated data. The empirical threshold values from Monte Carlo simulations for the Chi-square statistics and magnitude of the measures by item are obtained, based on 1000 simulations and α = 0.01 (α is considered to be 0.01 because DIF procedures are based on logistic regression, known to yield inflated Type-I error rates, especially when the groups differ substantially in the trait being measured [22, 23]). This is the unique feature of lordif package, which is not functionally available in other DIF detection approaches (interested readers can refer to Choi et al. [17]).

#### Analysis of cross-informants agreement

After using the DIF detection technique to evaluate the accuracy of the instrument, paired-sample t-test and intra-class correlation coefficient (ICC: as a measures of agreement) [24] were used to compare the parents and children’s grades and assess all dyads agreement in reporting children’s HRQoL, respectively. The mean difference was also determined and standardized by dividing the pooled standard deviation of both scores (effect size). In order to ascertain the magnitude of these differences, Cohen’s effect size was categorized as small (ES =|0.2|), medium (ES =|0.5|) and large (ES =|0.8|) [25]. The ICC values for agreement were also considered as poor (< 0.40), moderate (0.41–0.60), good (0.61–0.80) and excellent (> 0.81) [24]. In order to assess whether the observed subscale scores across daughter/son and mother/father reports were significantly affected by DIF items, we removed certain items with uniform DIF in all subscales. It is accepted that when the effect of an item with uniform DIF cannot be cancelled out by another uniform DIF item in the opposite direction, its effect can be transferred to the scale level. In this part of the analysis, data processing was carried out, using SPSS 18.0 [26].

## Results

The results of cross-informant consistency at both item and scale levels of PedsQL™ 4.0 are presented in the following part. First, mothers and fathers’ perceptions of their daughters and sons’ HRQoL are presented and analyzed at the item level of PedsQL™ questionnaire, by focusing on the effect of adolescence gender on the fathers and mothers’ report. Second, agreement between the informants was analyzed at the scale level of PedsQL^TM^4.0, by controlling the children’s gender.

### DIF analysis

Tables 2, 3, 4 and 5 present the results of the hybrid OLR/IRT model to detect DIF across the mothers and daughters, fathers and daughters, mothers and sons, and fathers and sons, respectively. To evaluate the possible confounding effect of the child’s gender, the following results *compared* the result of DIF analysis across father–child report *with* mothers–child report by considering the child’s gender.

**Table 2:** The results of the hybrid OLR/IRT DIF analysis across mother and daughter on the PedsQL™ 4.0 (Empirical threshold values from Monte Carlo simulations is also reported)

	Non-uniform				Uniform					
	*P*^a^	Threshold^b^	ΔR^2c^	Threshold^d^	*P*^a^	Threshold^b^	ΔR^2c^	Threshold^d^	CvBL^e^	Threshold^f^
*Physical health*										
5. Hard to take a bath	0.3156	0.0107	0.0018	0.0117	*0.0002*	0.0141	*0.0243*	0.0114	0.0135	0.0391
*Emotional functioning*										
3. Feel angry	0.8460	0.0105	0.0000	0.0039	*0.0000*	0.0094	*0.0115*	0.0040	*0.0270*	0.0092
5. Worry about what will happen	0.8531	0.0078	0.0000	0.0040	*0.0000*	0.0168	0.0097	0.0032	*0.0167*	0.0097
*Social functioning*										
1. Trouble getting along with peers	0.0576	0.0258	0.0027	0.0039	*0.0000*	0.0077	*0.0125*	0.0055	*0.0332*	0.0171
4. Doing things	*0.0029*	0.0327	*0.0080*	0.0046	0.0000	0.0110	0.0155	0.0063	0.0187	0.0188
5. Hard to play with others	0.2825	0.0255	0.0009	0.0038	*0.0000*	0.0068	*0.0472*	0.0057	*0.1236*	0.0178
*School functioning*										
1. Hard to concentrate	*0.0000*	0.0209	*0.0742*	0.0036	0.0186	0.0115	0.0037	0.0044	0.0042	0.0125
2. Forget things	*0.0003*	0.0188	*0.0084*	0.0037	0.1182	0.0069	0.0016	0.0048	0.0003	0.0116
3. Trouble schoolwork	0.4073	0.0188	0.0006	0.0060	*0.0000*	0.0069	*0.0467*	0.0069	*0.0937*	0.0209
4. Miss school-not well	*0.0080*	0.0092	*0.0069*	0.0067	0.8240	0.0046	0.0000	0.0082	0.0010	0.0211
5. Miss school-doctor appointment	*0.0022*	0.0162	*0.0085*	0.0055	0.0306	0.0140	0.0043	0.0056	0.0103	0.0205

Italic numbers represent the items showing uniform or non-uniform DIF

^a^*P *value of Chi-square test of difference between the Models 1 and 2, and Models 2 and 3 for testing uniform and non-uniform DIF, respectively

^b^Threshold values for the nominal α level associated with Chi-square test of difference between the Models 1 and 2, and Models 2 and 3

^c^*ΔR*^2^ is the *R*^2^ difference between the Models 1 and 2, and Models 2 and 3 for testing uniform and non-uniform DIF, respectively

^d^Threshold values for *ΔR*^2^, difference between the Models 1 and 2, and Models 2 and 3 for testing uniform and non-uniform DIF, respectively

^e^Crane, van Belle and Larson criterion—CvBL: |β1 (Model 1) − β1 (Model 2)/β1 (Model 1)|

^f^Threshold values of Crane, van Belle and Larson criterion—CvBL: |β1 (Model 1) − β1 (Model 2)/β1 (Model 1)|

**Table 3:** The results of the hybrid OLR/IRT DIF analysis across fathers and daughters on the PedsQL™ 4.0 (Empirical threshold values from Monte Carlo simulations is also reported)

	Non-uniform				Uniform					
	*P*^a^	Threshold^b^	ΔR^2c^	Threshold^d^	*P*^a^	Threshold^b^	ΔR^2c^	Threshold^d^	CvBL^e^	Threshold^f^
*Physical health*										
5. Hard to take a bath or shower	0.0749	0.0071	0.0052	0.0118	*0.0000*	0.0080	*0.0383*	0.0113	*0.0604*	0.0327
*Emotional functioning*										
2. Feel sad or blue	*0.0001*	0.0179	*0.0094*	0.0036	0.0000	0.0102	0.0355	0.0041	0.0935	0.0111
5. Worry about what will happen	0.5410	0.0154	0.0002	0.0034	*0.0000*	0.0080	*0.0317*	0.0040	*0.0668*	0.0115
*Social functioning*										
1. Trouble getting along with peers	0.0694	0.0311	0.0025	0.0037	*0.0000*	0.0122	*0.0156*	0.0049	*0.0241*	0.0131
3. Teased	0.2442	0.0188	0.0023	0.0097	*0.0000*	0.0086	*0.0286*	0.0122	*0.0633*	0.0277
4. Doing things other peers do School	*0.0000*	0.0172	*0.0189*	0.0061	0.0000	0.0138	0.0321	0.0067	0.0760	0.0176
5. Hard to keep up when play with others	0.2014	0.0237	0.0012	0.0040	*0.0000*	0.0063	*0.0408*	0.0059	*0.0883*	0.0154
*School functioning*										
1. Hard to concentrate	*0.0000*	0.0204	*0.0543*	0.0038	0.0850	0.0070	0.0020	0.0051	0.0014	0.0126
2. Forget things	*0.0000*	0.0186	*0.0176*	0.0039	0.0001	0.0089	0.0111	0.0048	0.0106	0.0152
3. Trouble keeping up with schoolwork	0.0525	0.0133	0.0032	0.0059	*0.0000*	0.0100	*0.0578*	0.0060	*0.1251*	0.0167

Italic numbers represent the items showing uniform or non-uniform DIF

^a^*P *value of Chi-square test of difference between the Models 1 and 2, and Models 2 and 3 for testing uniform and non-uniform DIF, respectively

^b^Threshold values for the nominal α level associated with Chi-square test of difference between the Models 1 and 2, and Models 2 and 3

^c^*ΔR*^2^ is the *R*^2^ difference between the Models 1 and 2, and Models 2 and 3 for testing uniform and non-uniform DIF, respectively

^d^Threshold values for *ΔR*^2^, difference between the Models 1 and 2, and Models 2 and 3 for testing uniform and non-uniform DIF, respectively

^e^Crane, van Belle and Larson criterion—CvBL: |β1 (Model 1) − β1 (Model 2)/β1 (Model 1)|

^f^Threshold values of Crane, van Belle and Larson criterion—CvBL: |β1 (Model 1) − β1 (Model 2)/β1 (Model 1)|

**Table 4:** The results of the hybrid OLR/IRT DIF analysis across Mothers and sons on the PedsQL™ 4.0 (Empirical threshold values from Monte Carlo simulations is also reported)

	Non-uniform				Uniform					
	*P*^a^	Threshold^b^	ΔR^2c^	Threshold^d^	*P*^a^	Threshold^b^	ΔR^2c^	Threshold^d^	CvBL^e^	Threshold^f^
*Physical health*										
2. Hard to run	0.4751	0.0117	0.0004	0.0051	0.0022	0.0067	0.0074	0.0058	0.0336	0.0282
5. Hard to take a bath or shower	0.1780	0.0109	0.0047	0.0179	0.0000	0.0044	0.0695	0.0213	0.0194	0.0619
*Emotional functioning*										
3. Feel angry	0.1464	0.0240	0.0013	0.0031	*0.0000*	0.0100	*0.0107*	0.0041	*0.0181*	0.0089
5. Worry about what will happen	*0.0000*	0.0147	*0.0177*	0.0035	0.0000	0.0147	0.0222	0.0036	0.0597	0.0098
*Social functioning*										
1. Trouble getting along with peers	0.5320	0.0201	0.0003	0.0044	*0.0000*	0.0065	*0.0341*	0.0061	*0.0905*	0.0188
3. Teased	0.1444	0.0108	0.0022	0.0071	*0.0000*	0.0071	*0.0174*	0.0077	0.0137	0.0238
5. Hard to keep up when play with others	*0.0026*	0.0155	*0.0066*	0.0044	0.0000	0.0072	0.0360	0.0056	0.0962	0.0231
*School functioning*										
3. Trouble keeping up with schoolwork	0.0126	0.0112	0.0047	0.0049	*0.0000*	0.0069	*0.0298*	0.0057	*0.0402*	0.0198
5. Miss school—doctor appointment	0.2566	0.0122	0.0012	0.0060	*0.0023*	0.0080	*0.0089*	0.0070	*0.0538*	0.0379

Italic numbers represent the items showing uniform or non-uniform DIF

^a^*P *value of Chi-square test of difference between the Models 1 and 2, and Models 2 and 3 for testing uniform and non-uniform DIF, respectively

^b^Threshold values for the nominal α level associated with Chi-square test of difference between the Models 1 and 2, and Models 2 and 3

^c^ΔR^2^ is the R^2^ difference between the Models 1 and 2, and Models 2 and 3 for testing uniform and non-uniform DIF, respectively

^d^Threshold values for ΔR^2^, difference between the Models 1 and 2, and Models 2 and 3 for testing uniform and non-uniform DIF, respectively

^e^Crane, van Belle and Larson criterion—CvBL: |β1 (Model 1) − β1 (Model 2)/β1 (Model 1)|

^f^Threshold values of Crane, van Belle and Larson criterion—CvBL: |β1 (Model 1) − β1 (Model 2)/β1 (Model 1)|

**Table 5:** The results of the hybrid OLR/IRT DIF analysis across fathers and sons on the PedsQL™ 4.0 (Empirical threshold values from Monte Carlo simulations is also reported)

	Non-uniform				Uniform					
	*P*^a^	Threshold^b^	ΔR^2c^	Threshold^d^	*P*^a^	Threshold^b^	ΔR^2c^	Threshold^d^	CvBL^e^	Threshold^f^
*Physical health*										
2. Hard to run	0.0336	0.0139	0.0035	0.0049	*0.0016*	0.0064	0.0077	0.0061	0.0345	0.0244
5. Hard to take a bath or shower	0.0524	0.0096	0.0095	0.0164	*0.0000*	0.0119	*0.0733*	0.0172	0.0114	0.0579
*Emotional functioning*										
5. Worry about what will happen	*0.0000*	0.0150	*0.0116*	0.0037	0.0000	0.0070	0.0310	0.0045	0.0642	0.0120
*Social functioning*										
1. Trouble getting along with peers	*0.0142*	0.0297	*0.0047*	0.0038	0.0000	0.0082	0.0351	0.0054	0.0745	0.0128
3. Teased	0.2351	0.0146	0.0015	0.0065	0.0000	0.0052	0.0327	0.0086	*0.0559*	0.0166
4. Doing things other peers do school	*0.0000*	0.0268	*0.0248*	0.0045	0.0141	0.0138	0.0054	0.0060	0.0096	0.0142
5. Hard to keep up when play with others	*0.0058*	0.0212	*0.0057*	0.0041	0.0000	0.0081	0.0170	0.0052	0.0264	0.0155
*School functioning*										
2. Forget things	0.0161	0.0210	*0.0042*	0.0038	0.0086	0.0063	*0.0050*	0.0055	0.0145	0.0183
3. Trouble keeping up with schoolwork	0.0001	0.0210	*0.0124*	0.0046	0.0000	0.0063	*0.0302*	0.0055	0.0451	0.0220

Italic numbers represent the items showing uniform or non-uniform DIF

^a^*P *value of Chi-square test of difference between the Models 1 and 2, and Models 2 and 3 for testing uniform and non-uniform DIF, respectively

^b^Threshold values for the nominal α level associated with Chi-square test of difference between the Models 1 and 2, and Models 2 and 3

^c^ΔR^2^ is the R^2^ difference between the Models 1 and 2, and Models 2 and 3 for testing uniform and non-uniform DIF, respectively

^d^Threshold values for ΔR^2^, difference between the Models 1 and 2, and Models 2 and 3 for testing uniform and non-uniform DIF, respectively

^e^Crane, van Belle and Larson criterion—CvBL: |β1 (Model 1) − β1 (Model 2)/β1 (Model 1)|

^f^Threshold values of Crane, van Belle and Larson criterion—CvBL: |β1 (Model 1) − β1 (Model 2)/β1 (Model 1)|

#### DIF analysis between mothers and daughters in compare to fathers and daughters

Comparison of the *P* values with threshold values for the nominal α level associated with Chi-square test of DIF analysis across mother and daughter (Table 2) indicated that 11 out of 23 items (47%) were flagged with DIF: one item in physical, two items in emotional, three items in social and all the items in school subscales. Amongst these items, six items (55%) exhibited uniform and five items (45%) non-uniform DIF (The uniform DIF items in the presence of the non-uniform DIF should be considered as non-uniform DIF items [20], e.g. item four in social subscale). For all six items with statistically significant uniform DIF, the differences in McFadden pseudo R^2^ (ΔR^2^) from Model 1 to Model 2 ranged from 0.0097 to 0.0472, which were greater than their own empirical criteria (i.e. all of them are practically important, except item 5 in the emotional subscale). Moreover, for the same six items with uniform DIF, the absolute proportionate β_1_ change effect size (Δβ_1_) ranged from 0.0135 to 0.1236, which were greater than their own empirical threshold values, except for item 5 in the physical subscale. Furthermore, for the four items with statistically significant non-uniform DIF, ΔR^2^ from Model 2 to Model 3 varied from 0.0069 to 0.0742, all of which were greater than the threshold values identified in Monte Carlo simulations.

The result of DIF analysis across fathers and daughters is shown in Table 3. As indicated by the results, 10 out of 23 items (43%) exhibited DIF; six of them (60%) were flagged uniform and four of them (40%) were non-uniform DIF, of which one item was in physical, two items in emotional, four items in social and three items in school functioning. Regarding the ΔR^2^ and Δβ_1_, all are practically important.

**Fig. 1:**
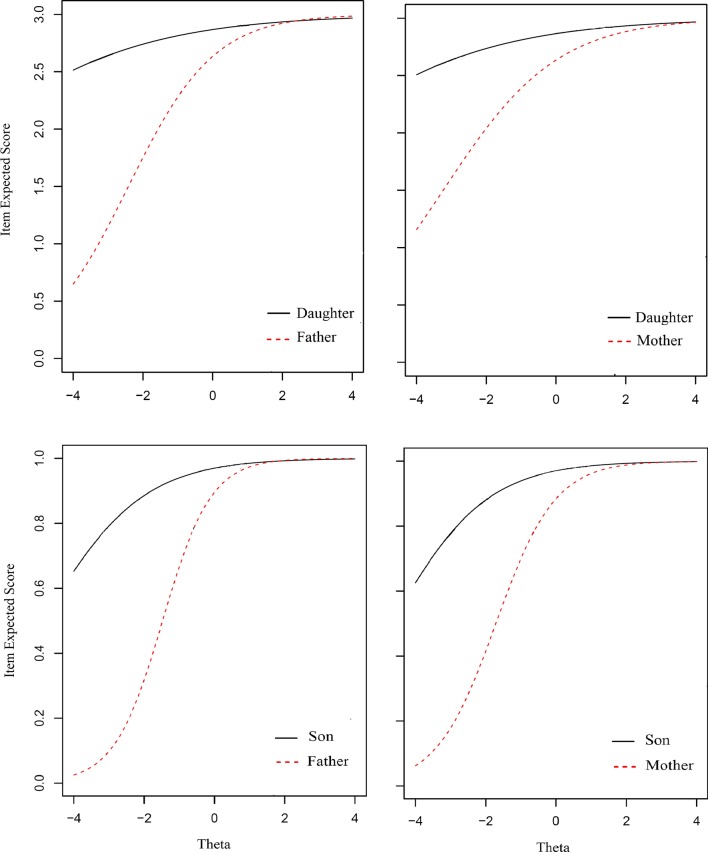
Comparison of father–daughter invariance to mother–daughter invariance (first row) and father–son invariance to mother–son invariance (second row) in item 5 in the physical subscale

Therefore, comparing the result of DIF analysis across fathers and daughters with the mothers and daughters indicated that the pattern of the number of DIF items in different subscales was almost similar to each other. This result is better represented graphically in the first row of Fig. 1, which shows that the expected score function for item 5 in physical subscale (as an example of a DIF item) exhibited the same direction in showing DIF between mothers and daughters, and fathers and daughters. Almost a similar result was obtained for the other DIF items, when comparing mother-report with father-report in rating their daughter.

Since it could be interesting for the readers to compare the pattern of DIF between mother/father–daughter to mother/father–son in item 5 in physical subscale, the graphical representation of the latter was also added to the Fig. 1 right here, according to reviewer suggestion.

#### DIF analysis between mothers and sons compared to fathers and sons

**Fig. 2:**
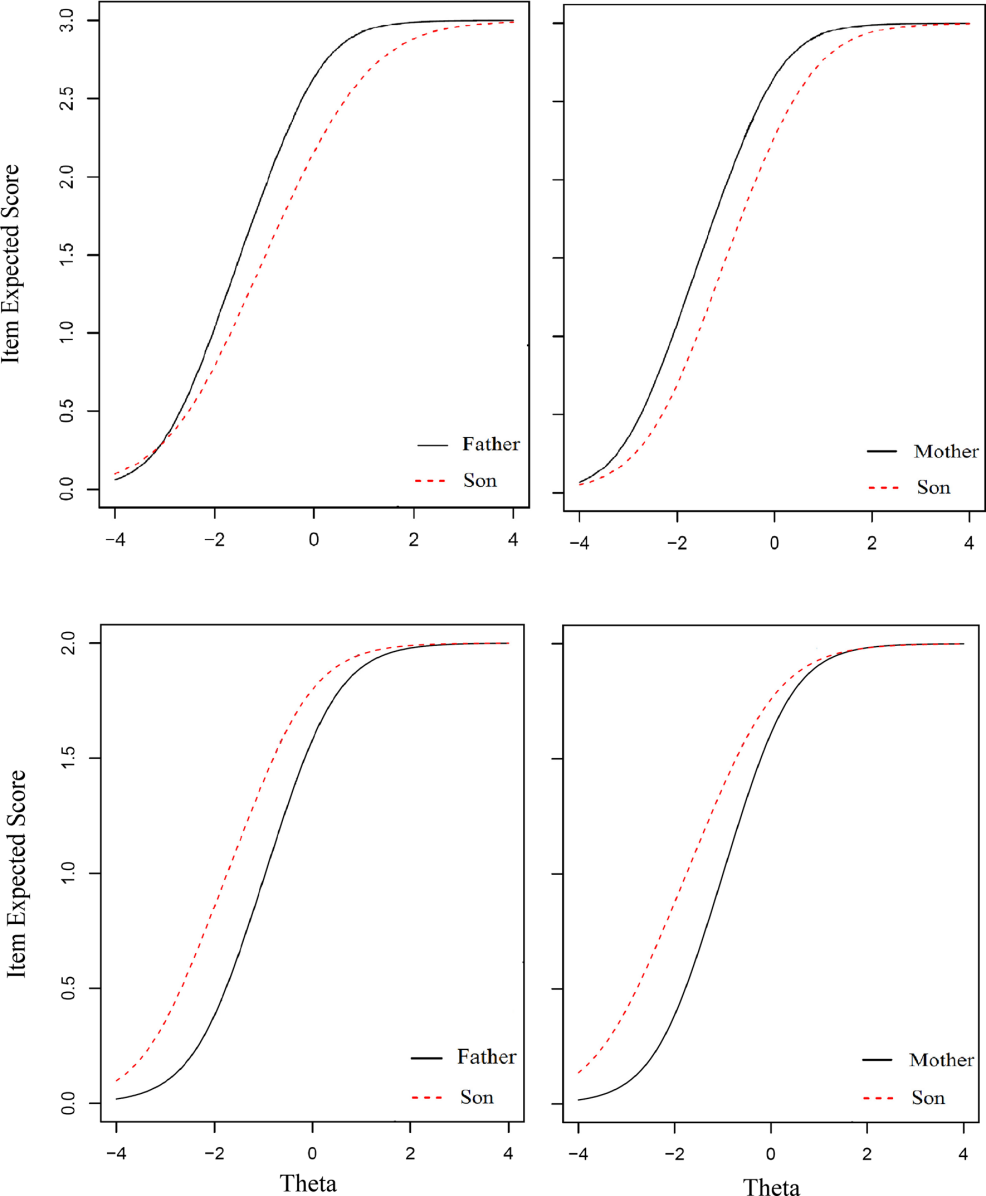
Graphical representation of father–son invariance and mother–son invariance in Item 1 (first row) and Item 3 (second row) in social subscale

Tables 4 and 5 present the results of DIF analysis across mothers and sons and fathers and sons, respectively. Although in both, 9 out of 23 items (39%) were flagged with DIF, the formation of DIF items and number of uniform and non-uniform DIF amongst several subscales was slightly different. It can be seen that amongst the mothers and sons, seven items (77%) exhibited uniform and two items (23%) revealed non-uniform DIF (Table 4), while it showed exactly a reverse pattern in the result of DIF analysis across fathers and sons (Table 5). To be more specific, in the former, two items in each of the physical, emotional and school subscales and three items in social functioning exhibited DIF, while two items in physical and school subscales, one item in emotional and four items in social showed DIF in the latter. Evaluating the magnitude of the measures, ΔR^2^ and Δβ_1_ indicated that all of them were practically important. It should be mentioned that in the DIF analysis the effect of items with uniform DIF can be cancelled out at the domain level by other uniform DIF items in the opposite direction. For example, as presented in Fig. 2, from the two items showing uniform DIF in the social subscale, item 1 showed DIF in one direction, whereas item 3 exhibited DIF in the opposite direction; hence, they canceled each other out (this condition is satisfied for both parents rating their sons HRQoL). The same result was obtained for items 3 and 5 in the emotional subscale. Accordingly, by comparing mother-to father-report in rating their sons, it indicated that although the pattern of DIF items was a bit different, in general most uniform DIF was cancelled out from the analysis.

#### Measure of cross-informants agreement

Table 6 shows the agreement of mothers and fathers individually with their daughters and sons with and without items with DIF. Within all dyads and based on ICC measures, small-to-moderate agreement was found in all the subscales. The highest agreement was found for physical health and the lowest for social functioning [both between mothers and daughters (ICC = 0.57 and ICC = 0.31, respectively)]. In general, the measure of concordance between mothers and children was observed to be greater than fathers and children in most subscales, regardless of the child’s gender.

**Table 6:** Mean Score, intra class correlation coefficients, for assessing parental agreement in rating child’s HRQOL in PedsQL™ 4.0 generic core scale

PedsQL™ 4.0 generic core scales	Mean ± (SD)	Effect size	ICC		Mean ± (SD)	Effect size	ICC
*Total*							
Mother	76.67 ± 13.90	0.14	0.55**	Father	78.06 + 13.50	0.03	0.52**
Daughter	78.58 ± 13.52			Daughter	78.54 + 13.55		
Mother	78.11 ± 13.41	0.24	0.50**	Father	79.40 + 14.06	0.15	0.46**
Son	81.20 ± 11.86			Son	81.27 + 11.81		
*Physical health*							
Mother	75.52 ± 17.10	0.13	0.57**	Father	77.26 + 16.53	0.008	0.54**
Daughter	77.43 ± 15.90			Daughter	77.37 ± 15.93		
Mother	79.91 ± 15.83	0.31	0.44**	Father	80.57 + 17.04	0.26	0.38**
Son	84.54 ± 12.77			Son	84.58 + 12.77		
*Emotional functioning*							
Mother	69.05 ± 19.02	0.06	0.48**	Father	73.30 ± 18.87	0.26	0.50**
Daughter	67.83 ± 21.66			Daughter	68.04 ± 21.31		
Mother	73.12 ± 18.06	0.01	0.50**	Father	77.10 ± 17.00	0.22	0.46**
Son	72.78 ± 19.11			Son	73.07 ± 18.90		
*Social functioning*							
Mother	84.82 ± 15.48	0.17	0.43**	Father	84.71 ± 15.55	0.16	0.36**
Daughter	87.66 ± 16.25			Daughter	87.60 ± 16.31		
Mother	83.64 ± 15.47	0.08	0.31**	Father	82.64 ± 17.24	0.12	0.33**
Son	85.05 ± 16.25			Son	85.10 ± 15.55		
*School functioning*							
Mother	79.37 ± 17.39	0.18	0.43**	Father	79.42 ± 17.79	0.18	0.38**
Daughter	82.17 ± 14.43			Daughter	82.21 ± 14.46		
Mother	77.19 ± 17.31	0.18	0.48**	Father	78.02 ± 17.26	0.14	0.39**
Son	80.21 ± 15.87			Son	80.37 ± 15.87		
*Total*^a^							
Mother	76.81 ± 14.89	0.03	0.56**	Father	77.62 ± 13.68	0.06	0.57**
Daughter	77.25 ± 15.70			Daughter	76.92 ± 14.27		
Mother	78.44 ± 16.21	0.20	0.44**	Father	79.54 ± 13.54	0.08	0.48**
Son	81.37 ± 12.84			Son	80.64 ± 12.48		
*Physical health*^a^							
Mother	73.83 ± 18.23	0.05	0.60**	Father	75.25 ± 17.41	0.05	0.58**
Daughter	74.78 ± 17.82			Daughter	74.31 + 17.45		
Mother	78.43 ± 16.32	0.27	0.44**	Father	79.50 ± 16.74	0.20	0.36**
Son	82.55 ± 14.52			Son	82.62 ± 14.34		
*Emotional functioning*^a^							
Mother	74.80 ± 19.67	0.01	0.47**	Father	73.50 ± 19.57	0.06	0.47**
Daughter	74.31 ± 21.79			Daughter	72.30 ± 18.67		
Mother	79.57 ± 16.57	0.02	0.46**	Father	77.78 ± 16.31	0.11	0.43**
Son	79.93 ± 17.29			Son	76.06 ± 17.45		
*Social functioning*^a^							
Mother	91.28 + 15.27	0.05	0.39**	Father	88.33 ± 20.55	0.04	0.17**
Daughter	90.37 + 16.97			Daughter	89.13 ± 21.18		
Mother	88.16 ± 16.25	0.28	0.34**	Father	80.61 ± 18.01	0.03	0.32**
Son	84.77 ± 17.02			Son	81.34 ± 21.30		
*School functioning*^a^							
Mother	All the items showed DIF			Father	86.60 ± 18.21	0.06	0.48**
Daughter	No corrected score existed			Daughter	85.54 ± 16.98		
Mother	76.04 ± 17.07	0.11	0.48**	Father	87.57 ± 19.87	0.06	0.37**
Son	77.96 ± 17.17			Son	86.31 ± 21.02		

0 = Almost always, 25 = Often, 50 = Sometimes, 75 = Almost never, 100 = Never

*P ≤ 0.05; **P ≤ 0.0001; *CI* confidence interval, *SD* standard deviation, *ICC* intra-class correlation

^a^Score corrected for DIF items

Also listed in Table 6 are the means and standard deviations (SD) of mothers and fathers and their children scores, and the related effect size (ES). Although the mean score of the parents’ report was significantly different from their children in a few subscales, all the Cohen’s effect sizes were negligible. These findings reveal that fathers and mothers were not that different when it came to rating their daughters and sons, and both tended to report slightly the worst HRQoL than their child, except for emotional functioning. It should be mentioned that the result of cross-informant agreement did not change significantly before and after correction for DIF items (Table 6).

## Discussion

This is the first study investigating the effect of children’s gender on father and mother’s reports of their children’s HRQoL at both item and scale levels of PedsQLTM4.0 questionnaire. The results were unique, due to the integration of mothers and fathers’ views on daughters and sons’ HRQoL. Assessing DIF across mother–daughter, father–daughter, mother–son and father–son dyads revealed that although parents and their children perceived the meaning of several items of PedsQL^TM^4.0 instrument differently, the pattern of fathers and mothers’ report did not vary much across daughters and sons. In other words, the Persian version of PedsQLTM4.0 showed that the child’s gender was not a confounding factor when mothers and fathers reported their daughters and sons’ HRQoL.

In our previous study, it was shown that in the proxy version of PedsQLTM4.0, parents’ gender was not a confounding factor in reporting the child’s HRQoL [7]. The present study revealed that the child’s gender did not affect the results of parents’ reports regarding their children’s HRQoL. Although the children and their parents interpreted several items differently, taking the pattern of DIF items across the father–son, father–daughter, mother–son and mother–daughter into account (e.g. Figs. 1, 2), in PedsQL^TM^4.0, the parents and children’s gender was not an effective confounder when assessing the children’s HRQoL.

As far as we know, there is no similar study to compare our findings directly with them. In the closest study, the measurement invariance of the other pediatric HRQoL instruments (KIDSCREEN-27) across the son–parent and daughter–parent dyads was evaluated [8]. Although this report highlights the importance of taking the child’s gender into account when evaluating the measurement invariance, they noticed that this assertion should not be definite, without knowing the parent’s gender.

The result of parental evaluation of the child’s HRQoL at the scale level of PedsQL^TM^4.0 revealed a small to moderate level of agreement across the parents and children’s reports in all subscales (ICC = 0.31–0.57). It should be mentioned that the degree of parental agreement was a little different across the daughters and sons; although both fathers and mothers had a tendency to underestimate their children’s general HRQoL (except for emotional functioning which was overestimated), both parents had greater agreement with their daughters, and also father–son agreement was the lowest in all domains. This finding could be due to the fact that boys, as compared to girls, tend to be more independent in their activities [27]. In this study, a greater degree of agreement was detected between children and their mothers, especially girls, who see their mothers as their confidant, and this could be the result of the parents’ distinct roles in a family. In most cultures, including Iran, fathers are the providers while mothers are involved in rearing and raising their children. In a recent systematic review, Hemmingsson et al. assessed all studies related to the parent–child agreement in HRQoL research [28]. Despite showing small to moderate level of agreement, they could not reach consistent results, concerning whether or not the parent–child agreement was related to their children’s gender. For example, two studies found higher parent–child agreement in daughters [29, 30], which is in line with the current findings. In contrast, Carlston and Ogles showed greater disagreements between the daughters and parents, while the sons and parents exhibited more pervasive but less severe discrepancies [31]. Buck et al. also found that parents exaggerated their daughter’s overall HRQoL on the PedsQL questionnaire of psychosocial functioning, but they understated their sons [32]. In several aspects, this finding was in contrast with our results, which might be due to the differences in the study design and the statistical methods used for data analysis.

From a methodological point of view, measurement invariance of the PedsQL^TM^4.0 across the informants was assessed, using hybrid OLR/IRT model, through lordif, a powerful freeware package in R software for DIF detection [17]. One unique feature of this platform is the ability to detect DIF based on Type-I error rates which is empirically found in the simulated data. That is, for example, when we used the McFadden pseudo-R^2^ to quantify the magnitude of DIF, the values might vary from item to item, depending on the distribution within each response category and the number of response categories [19]. Accordingly, using a single threshold could result in varying powers across items to detect DIF [33]. Hence, simulations can help to inform the choice of sensible thresholds. In other words, if a single threshold is to be used across all items, it should be set above the highest value identified in simulations. For instance, the maximum McFadden pseudo-R^2^ in Table 2 was 0.0189; thus, a rational lower bound that could avoid Type-I errors might be 0.02, which interestingly corresponds with a non-negligible (i.e. small) Cohen effect size [25].

This study had a number of limitations that has to be considered before drawing any conclusion. First, in the present study, the majority of the participants were parents and children of apparently healthy population; if children or parents had a serious chronic illness, cross-informant agreement could have been affected. For example, in adolescents with significant health conditions, fathers and mothers attended to the daily functioning of their children. It seems that, in Iran, mothers, as compared with fathers, are more concerned about their children’s health; thus, it is unclear to what extent a child’s health status could influence the results of DIF analysis across fathers/mothers and daughters/sons. As a second limitation, the current study was limited to the adolescents aged 13–17 years-old since the fathers and mothers’ item response patterns was likely to be biased for samples that combine younger children and adolescents. Given the amount of time that adolescence, especially boys, spend away from home, agreement across father/mother and son/daughter might be potentially attenuated and the results of DIF analysis is confounded. Therefore, the results of this study cannot be generalized to children younger than 13 years. A third limitation arises from the point that the hybrid IRT/OLR models were conducted separately in each domain for evaluating DIF items. Nonetheless, considering multidimensional approaches for analyzing multidimensional PRO instrument, such as PedsQL^TM^ 4.0, could be much better in dealing with correlation amongst subscales and might principally change our results [34–36]. Further studies are warranted to identify the possible effect of multidimensional analysis in exploring DIF items. Although the potential dependency between parents group and children leads to the fourth limitation of this study, no simulation-based study so far has extended the iterative hybrid OLR/IRT approach for longitudinal data which could be much better handling dependency amongst the groups and controlling its possible effect on DIF detection [37, 38]. However, some other DIF detection techniques were introduced which could deal with this problem and model the between groups covariance. The actor–partner interdependence models [39, 40] and the longitudinal factor analysis based-models [41], which are tested measurement invariance over the time, are among these methods. Nonetheless, none of these methods could provide a simulation-based mechanism to evaluate statistical criteria for detecting DIF. Therefore, improving the longitudinal version of iterative hybrid OLR/IRT approach with Monte Carlo simulation could be considered for the future studies. The fifth limitation of the study arises from the fact that 40% of students did not take the questionnaires back to the research team. Since no socioeconomic indicators were available for non-participant students, we could not evaluate the potential enrollment bias. Finally, further research should consider these limitations and try to expand the findings to other pediatric HRQoL measures, such as KIDSCREEN-27 and KINDL, in order to develop a more reliable assessment tool for parent–child agreement studies in different cultures.

## Conclusion

In conclusion, this study revealed that although fathers/mothers and daughters/sons perceived the meaning of PedsQL™ 4.0 items differently, the pattern of the fathers and mothers’ report did not vary much across the daughters and sons. In the Persian version of PedsQLTM4.0, the child’s gender was not a confounding factor when the parents reported their daughters and sons’ HRQoL. This indicates that the mothers and fathers’ scores in reporting their children’s HRQoL are comparable without taking the child’s gender into account, suggesting that in Iran paternal proxy-reports can be included in the maternal proxy-reports, and the reports can be combined without considering the children’s gender.

## Data Availability

The datasets analyzed during the current study available from the corresponding author on reasonable request.

## Abbreviations

HRQoL: Health related quality of life
DIF: Differential item functioning
OLR: Ordinal logistic regression
IRT: Item response theory
GRM: Graded response model
